# Effect of an Anterior Cruciate Ligament Rupture on Knee Proprioception Within 2 Years After Conservative and Operative Treatment: A Systematic Review with Meta-Analysis

**DOI:** 10.1007/s40279-021-01600-z

**Published:** 2021-12-02

**Authors:** John Dick Fleming, Ramona Ritzmann, Christoph Centner

**Affiliations:** 1 Department of Sport and Sport Science, University of Freiburg, Schwarzwaldstraße 175, 79117 Freiburg, Germany; 2 Praxisklinik Rennbahn, Muttenz, Switzerland

## Abstract

**Background:**

The anterior cruciate ligament (ACL) plays a major role in knee proprioception and is thus responsible for maintaining knee joint stability and functionality. The available evidence suggests that ACL reconstruction diminishes somatosensory feedback and proprioceptive functioning, which are vital for adequate joint positioning and movement control.

**Objective:**

The aim of this systematic review and meta-analysis was to investigate the effect of an ACL rupture on knee proprioception after arthroscopic ACL repair surgery or conservative treatment.

**Methods:**

A systematic review with meta-analysis was conducted according to the Preferred Reporting Guidelines for Systematic Reviews and Meta-Analyses (PRISMA) guidelines. The literature search was performed in the following databases from inception to 10th October 2020: PubMed, Web of Science, SPORTDiscus, Cochrane Library and Scopus. Randomized and non-randomized studies that evaluated proprioception using the joint position sense (JPS) and threshold to detection of passive motion (TTDPM) techniques at 15°–30° knee flexion with an external healthy control group in a time period between 6 and 24 months post injury or operation were included in the analysis.

**Results:**

In total, 4857 studies were identified, from which 11 were included in the final quantitative analysis. The results demonstrated that proprioception after arthroscopic ACL repair surgery was significantly lower than in the healthy control group (JPS: standardized mean difference [SMD] 0.57, 95% confidence interval [CI] 0.27–0.87, *p* < 0.01, *n* = 6 studies; TTDPM: SMD 0.77, 95% CI 0.20–1.34, *p* < 0.01, *n* = 4 studies). There were no significant differences in proprioception between the conservative treatment group and the healthy control group (JPS: SMD 0.57, 95% CI − 0.69 to 1.84, *p* = 0.37, *n* = 4 studies; TTDPM: SMD 0.82, 95% CI − 0.02 to 1.65, *p* = 0.05, *n* = 2 studies), although measures for TTDPM were close to statistical significance.

**Conclusion:**

The findings of the present systematic review and meta-analysis revealed that knee proprioception is persistently compromised 6–24 months following surgical treatment of ACL tears compared with healthy controls. The reduced kinesthetic awareness after ACL surgery is of high relevance for optimizing individual treatment plans in these patients. As the current literature is still scarce about the exact underlying mechanisms, further research is needed.

**Trial Registration:**

The present systematic review was registered in PROSPERO (CRD42021198617).

**Supplementary Information:**

The online version contains supplementary material available at 10.1007/s40279-021-01600-z.

## Key Points

**Table:** 

We found high-level evidence suggesting that proprioceptive deficits are present following surgical anterior cruciate ligament reconstruction.
Although no significant deficits were detected following conservative approaches, we observed considerable heterogeneity between these studies that needs to be taken into account when interpreting the findings for conservative treatments.

## Introduction

The anterior cruciate ligament (ACL) plays a major role in maintaining knee joint stability because it contributes to both functionality and the mechanical congruence of the lateral and medial tibiofemoral joints. Based on its anatomical position, it resists the anterior tibial translation and rotational load [1]. Furthermore, knee ligaments are rich in sensory innervation, which allows them to be closely integrated in neural reflex pathways [2, 3]. Throughout abnormal strain, stimulation of mechanoreceptors in the ACL initiates different types of reflex responses through the neural arc to secure the arthrokinematics of the joint with an adequate muscle contraction [4, 5].

In sports, an ACL tear is one of the most common injuries, occurring mainly in pivoting high-load sports such as soccer, basketball and alpine ski [1]. The main focus of either an operative or conservative treatment and the following rehabilitation program is to therefore restore the stability and kinematics of the joint to ensure a safe return to sport. Nevertheless, studies indicate that of 82% of the patients who return to sport, only 63% compete at their original level of competition [6]. This is accompanied by an increased risk of recurrences in the first 2 years post injury [7] and the long-term consequences of developing knee osteoarthritis [8].

One cause of the increased risk of re-injury and performance declines is attributed to the diminished proprioception after an ACL rupture [9]. The native intact ACL contains mechanoreceptors that detect changes in direction of movement, changes in acceleration, speed, tension and an estimate of the joint position [10, 11]. The ACL does not heal when torn, and surgical reconstruction after a complete ACL tear using tendinous allografts or autografts does not allow for the re-innervation of mechanoreceptors [12]. A key factor in persistent functional instability after ACL tears is therefore experiencing kinesthetic deficiency and altered neuromuscular function secondary to a diminished somatosensory feedback [10, 11]. Proprioceptive information to accurately regulate the neuromuscular control is missing and may account for an increased risk of re-injury and coordination deficits when high performance is required. Methodological approaches to assess proprioception include the detection and reproduction of angular position, sense of tension, or effort [13]. Generally, the two most common protocols to reliably assess knee proprioception are the joint position sense (JPS) and threshold to detect passive motion (TTDPM) measures [14, 15]. JPS is assessed by measuring the reproduction of passive angular positioning [15]. TTDPM evaluates the ability of individuals to detect the onset of passive movement [16].

Although older previous systematic reviews exist [17, 18], which aimed to elucidate the effects of ACL injury on knee proprioception, both reviews included heterogeneous studies that limit the validity and significance of their conclusions. Heterogeneity arose from comparing studies that assessed broad time points extending from 8 to 60 months following ACL injury [18] or that focused on paradigms with different joint angles (e.g., 15° vs. 75° knee flexion [17]) in which ligamentous tension is not necessarily experienced and kinaesthesia is of negligible importance [19, 20]. Lastly, Relph and colleagues [17] included single study populations multiple times in their quantitative analyses, which inherently increases the statistical weighting of that specific population and thus potentially biases the results.

Against this background and the fact that numerous studies have recently been published on this topic, it is highly relevant to systematically re-investigate the effects of ACL injury on proprioception with strict inclusion criteria in order to ensure homogeneity between studies and thus allow a conclusive statement of clinical relevance. For that purpose, the present systematic review and meta-analysis aims to systematically consider several biasing factors, such as different timepoint assessments, various knee angle measurements, and the use of different autografts and allografts within the methodological approach. Subsequently, the clinical relevance will be determined by interpreting the calculated effect sizes.

## Methods

### Protocol and Registration

The present systematic review includes a meta-analytic approach and was completed in accordance with the Preferred Reporting Items for Systematic Reviews and Meta-Analyses (PRISMA) guidelines [21]. Therefore, a review protocol was prospectively elaborated and registered on PROSPERO (CRD42021198617).

### Search Strategy

To identify relevant studies, a literature search was performed in the following electronic databases from inception to 10 October 2020: PubMed, Web of Science, Scopus, SPORTDiscus and Cochrane Library. The search string consisted of synonyms from the field of ACL injury connected with synonyms for the topic of proprioception. Databases were searched without restrictions (“All field” search) and the final search string was:

(“Joint Position Sense” OR “TTDPM” OR Proprioception OR active angle reproduction OR Threshold to detect passive motion OR passive angle reproduction OR kinesthes* OR somatosensory* OR mechanorecept* OR “balance”) AND (“Anterior cruciate ligament” OR “ACL rupture” OR “ACL reconstruction” OR “ACL injury” OR “ACL deficient” OR “knee injury” OR “knee joint” OR “ACL replacement”).

**Fig. 1:**
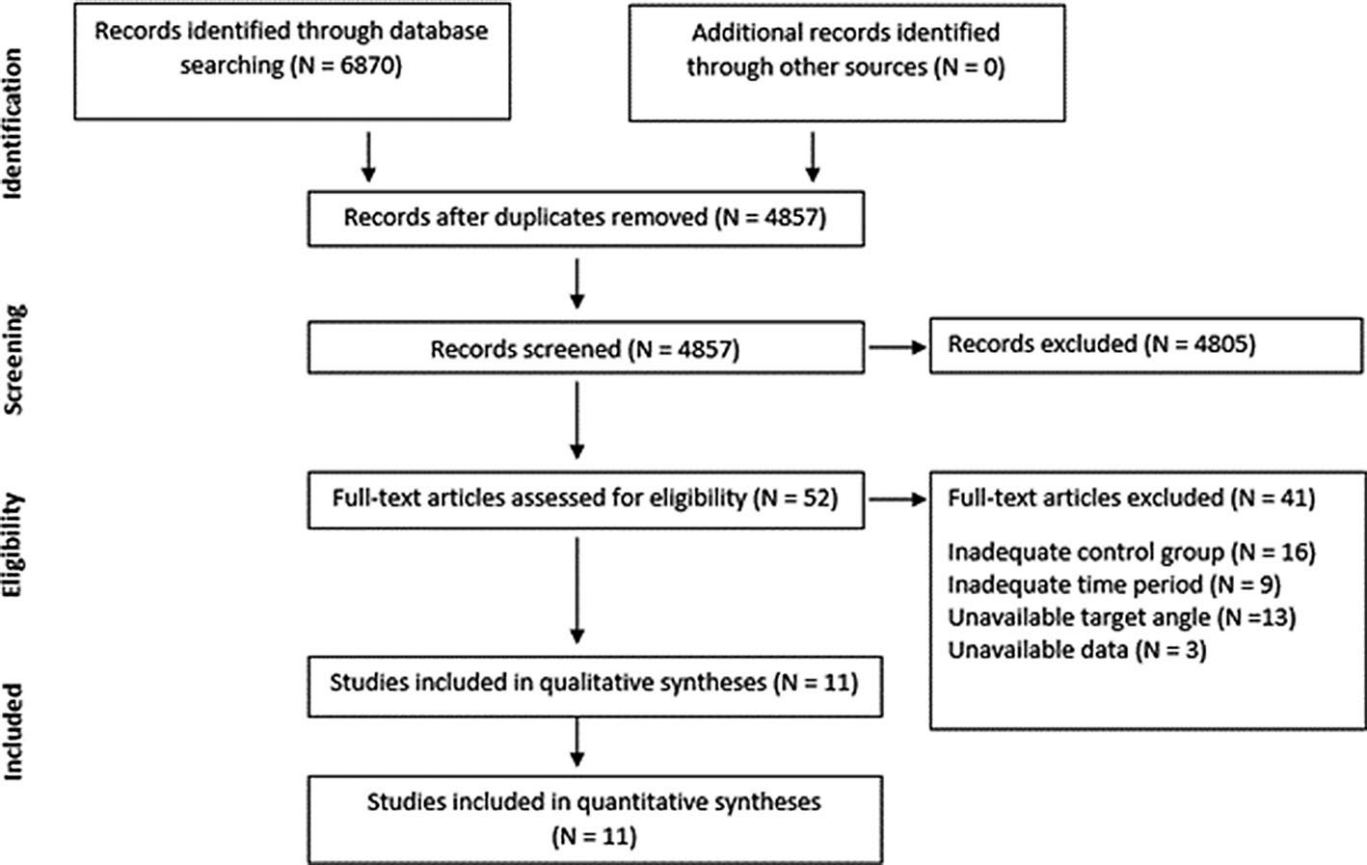
Study selection process

Additionally, the reference lists of eligible articles were systematically screened for further eligible papers [22]. All studies including title and abstract were exported to a citation manager and duplicates were removed before further processing (for the search process, see Fig. 1).

### Study Selection and Eligibility Criteria

To assess eligibility, two reviewers independently evaluated the results by screening the title and abstracts. In cases of relevant titles, the full text was assessed. The inclusion criteria included (1) complete primary ACL rupture; (2) no additional knee pathology; (3) the therapeutic treatment included either surgery or a conservative approach; and (4) comparison with a healthy control (HC) group. Comparing against HCs is crucial given that evidence suggests that the proprioception of the contralateral intact knee can also be compromised following an ACL tear [18, 23]. Re-ruptures and incomplete ruptures were excluded to ensure an homogenous population with comparable sensorimotor prerequisites. Lastly, only studies with proprioception assessments between 0° and 30° knee angle were included in order to avoid inhomogeneity of studies. The reason for focusing on this angle range was that the ACL is subjected to the highest stress in terminal knee positions [24, 25].

Studies were excluded if their study design incorporated a specific treatment (e.g., knee orthosis, proprioceptive training, etc.). Furthermore, studies were not included if proprioception was measured later than 24 months post trauma, since this has been postulated to be an important time span for return to sport and increased re-injury rates [7, 26, 27]. Finally, studies with weak quality (score ≤ 14 on the Downs and Black Checklist [28]) were excluded from the qualitative and quantitative analyses.

The identified studies were subsequently grouped regarding treatment (operative vs. conservative) and quantitative analyses were conducted considering the reliable and accurate measurement techniques of proprioception: JPS and TTDPM.

### Data Extraction and Collection

After an initial screening, the following information was extracted from relevant articles: (1) population characteristics; (2) measurement timepoints; (3) methodological approach; and (4) main findings.

During data extraction, only timepoints within the first 24 months following the ACL injury were considered. Since some studies reported several timepoints within this period, an a priori prioritization list was developed [29, 30] and the timepoints were considered in the following order: 24 months > 12 months > 6 months > 3 months. Additionally, target angles during proprioception measurements were prioritized from 15° to 30°. The reason for this prioritization was that the injury mechanism has previously been associated with a knee angle around 15° [31–33]. Furthermore, measurements in the direction of knee extension were considered first compared with flexion, when measurements in both directions were available. If only knee flexion values were available, these were also included in the analysis. Lastly, if both allograft and autografts were included, autografts were prioritized since these are more frequently used in ACL surgery [1]. Within autografts, the following order was chosen: (1) bone-to-bone patellar tendon autograft; (2) semitendinosus tendon autograft; (3) quadriceps tendons autograft.

In cases of unavailable raw data, the corresponding author of the manuscript was contacted. If the respective authors were non-responsive, data were extrapolated from figures using ImageJ software (National Institutes of Health, Bethesda, MD, USA). If extrapolation from figures was not possible, data were discussed within the qualitative analyses but not within the quantitative evaluation.

All data were independently extracted and screened by two researchers (JF and CC). In the case of disagreement, consensus was found in one of the regular discussion meetings.

### Study Quality

Study quality was assessed using the Downs and Black Checklist [28, 34]. The checklist is used to evaluate randomized and non-randomized studies and consists of 27 items covering the following quality characteristics: reporting, external validity, internal validity (bias and confounding variables) and power. The total score is categorized as follows: very good (26–28 points), good (20–25 points), moderate (15–19 points), or weak (0–14 points) [34].

### Risk-of-Bias Assessment

Risk of bias within each study was determined using the JBI Critical Appraisal Checklist for analytical cross-sectional trials [35–37]. This tool comprises a total of eight items and two researchers (JF and CC) independently rated each item as ‘yes’, ‘no’, ‘unclear’ or ‘not applicable’ as per protocol [35]. The studies were rated as low risk of bias if more than 70% of the items were applicable (‘yes’ answer). In cases of 50–69% applicable items, a moderate risk of bias was assumed, and with < 49% applicable items, a high risk of bias was assumed [38].

### Synthesis of Results and Statistical Approach

For the quantitative meta-analytical combination, the standardized mean difference (SMD) was calculated from the primary studies. The SMD was computed by dividing the mean difference by the pooled standard deviation [39]. Therefore, mean, sample size, and standard deviation were extracted. If mean or standard deviation from a study were not reported, these were respectively estimated from the median or standard error and confidence intervals (CIs) according to the Cochrane Handbook of Systematic Reviews [39]. All meta-analyses were performed using a random-effects model with inverse variance weighting, and forest plots were subsequently created. To examine inconsistency and heterogeneity across studies, the *I*-square method was used. *I*-square was calculated as [(Chi-square statistic − degrees of freedom)/Chi-square statistic × 100%] [39]. With respect to previous interpretation guidelines [39], *I*-square was interpreted as follows: 0–40% representing low heterogeneity, 30–60% representing moderate heterogeneity, 50–90% representing substantial heterogeneity, and 75–100% representing considerable heterogeneity. Lastly, a qualitative analysis of proprioception following ACL reconstructed (ACLR) and ACL deficient (ACLD) was conducted taking the different methodologies of proprioception assessment into account.

## Results

### Study Selection

In total, 4857 studies were identified throughout the literature search, with 11 finally included in this systematic review with meta-analysis. We assessed the full-texts from 52 studies, from which 41 were excluded due to an inadequate control group (*n* = 16), inadequate time period (*n* = 9), unavailable target angle (*n* = 13), or unavailable data (*n* = 3). The remaining 11 studies were then assigned to their treatment (operative or conservative) and, for quantitative analyses, again divided (and independently analyzed) into studies using JPS and TTDPM as measures of proprioception.

### Study Characteristics

Within the included studies comparing patients with a medical history of surgical ACL reconstruction with HCs, six studies quantified knee proprioception using JPS [40–45] and four studies by means of TTDPM [41, 42, 46, 47]. In three studies, JPS was estimated in a seating position [40, 44, 45]; three studies evaluated JPS in a standing [43], supine [42], or lateral recumbent position [41], respectively. Similarly, TTDPM was measured in the seated [46, 47], supine [42], and lateral recumbent positions [41]. The mean time between injury and assessment (which was included in the present quantitative analysis) in the comparison between ACLR and HCs was 14 months (see Tables 1 and  2).

**Table 1:** Study characteristics of the ACLR group—JPS (*n* = 6)

Study	Groups	Months after injury	JPS set-up	Starting and target angle
Fremerey et al. [40]	ACLR = 20 HC = 20	3, 6	PAR Seated 0.5°/s	Start at 0° TF Target angle: 0–20°, 40–60°, 80–100°
Roberts et al. [41]	ACLR = 20 HC = 19	24	AAR Lateral recumbent position	Start at 30° TF and 60° TE Target angle: 60° (TF) and 30° (TE)
Bonfim et al. [42]	ACLR = 10 HC = 10	18	Verbal, external goniometer Supine position	Start at 0° TF Target angle: 0°, 15°, 30°, 45°, 60°
Mir et al. [43]	ACLR = 12 HC = 12	11	AAR Standing	Start at 0° TF and 60° TE Target angle: 30°
Zhou et al. [44]	ACLR = 36 HC = 13	6	PAR Seated 2°/s	Start at 0° TF Target angle: 0–20°, 40–60°, 80–100°
San Martín-Mohr et al. [45]	ACLR = 30 HC = 27	7.77 ± 2.28	AAR Seated	Start at 90° TE Target angle: 0–30°, 30–60°, 60–90°

*ACLR* anterior cruciate ligament reconstructed, *HC* healthy control, *JPS* joint position sense, *PAR* passive angle reproduction, *AAR* active angle reproduction, *TF* towards flexion, *TE* towards extension

**Table 2:** Study characteristics of the ACLR group—TTDPM (*n* = 4)

Study	Groups	Months after injury	TTDPM set-up	Starting angle and direction of movement
Roberts et al. [41]	ACLR = 20 HC = 19	24	Lateral recumbent position 0.5°/s	Start at 20° and 40° TF and TE
Bonfim et al. [42]	ACLR = 10 HC = 10	18	Supine position 0.5°/s	Start TF at 0°, 15°, 30°, 45°, 60° Start TE at 15°, 30°, 45°, 60°
Ozenci et al. [46]	ACLR = 20 HC = 20	16.5 ± 5.5	Seated 1°/s	Start at 15° TE and TF
Laboute et al. [47]	ACLR = 32 HC = 32	6	Seated 4°/s	Start at 15° TF

*ACLR* anterior cruciate ligament reconstructed, *HC* healthy control, *TTDPM* threshold to determine passive motion, *TF* towards flexion, *TE* towards extension

Within studies comparing ACL-deficient patients and HCs, four studies assessed JPS [40, 48–50] and two studies assessed TTDPM [46, 48]. Positions for JPS included the seated [40, 49, 50] and lateral recumbent positions [48]. Studies using TTDPM investigated knee proprioception, also in the seated [46] and lateral recumbent positions [48] (Tables 3 and  4).

**Table 3:** Study characteristics of the ACLD group—JPS (*n* = 4)

Study	Groups	Months after injury	JPS set-up	Starting and target angle
Fridén et al. [48]	ACLD = 16 HC = 19	1, 2, 4, 8	AAR Lateral recumbent position	Start at 30° TF and 60° TE Target angle: 30°
Fremerey et al. [40]	ACLD = 20 HC = 20	12.4 ± 3.7	PAR Seated 0.5°/s	Start at 0° TF Target angle: 0–20°, 40–60°, 80–100°
Relph and Herrington [49]	ACLD = 20 HC = 20	11 ± 2	AAR Seated	Start at 0° TF Target angle: 10–30°
Zult et al. [50]	ACLD = 32 HC = 20	7	AAR Seated	Start at 90° TE Target angle: 15°, 30°, 45°, 60°

*ACLD* anterior cruciate ligament deficient, *HC* healthy control, *JPS* joint position sense, *PAR* passive angle reproduction, *AAR* active angle reproduction, *TF* towards flexion, *TE* towards extension

**Table 4:** Study characteristics of the ACLD group—TTDPM (*n* = 2)

Study	Groups	Months after injury	TTDPM set-up	Starting angle and direction of movement
Fridén et al. [48]	ACLD = 16 HC = 19	1, 2, 4, 8	Lateral recumbent position 0.5°/s	Start at 20° TE and TF Start at 40° TE and TF
Ozenci et al. [46]	ACLD = 20 HC = 20	12.5 ± 3.6	Seated 1°/s	Start at 15° TE and TF

*ACLD* anterior cruciate ligament deficient, *HC* healthy control, *TTDPM* threshold to determine passive motion, *TF* towards flexion, *TE* towards extension

### Anterior Cruciate Ligament Reconstructed (ACLR) Versus Healthy Controls (HCs)

The results of the ACLR group compared with the HC group revealed significant differences in both measures for proprioception (JPS and TTDPM).

**Fig. 2:**
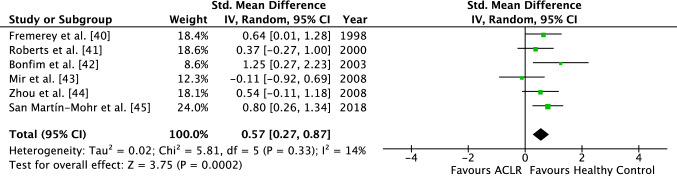
Forest plot of the ACLR group demonstrating the JPS between the operated group and the healthy control group. *ACLR* anterior cruciate ligament reconstructed, *JPS* joint position sense, *Std.* standardized, *IV* inverse variance, *CI* confidence interval, *df* degrees of freedom

In total, six studies evaluated JPS and four studies analyzed TTDPM. The analysis for the JPS demonstrated an SMD of 0.57 (95% CI 0.27–0.87), favoring the HC group (Fig. 2). On average, the mean angle of error was therefore significantly higher for the ACLR group compared with the HC group (*Z* = 3.75, *p* < 0.01). Study heterogeneity was low (*I*^2^ = 14%) and not statistically significant (*p* = 0.33).

**Fig. 3:**
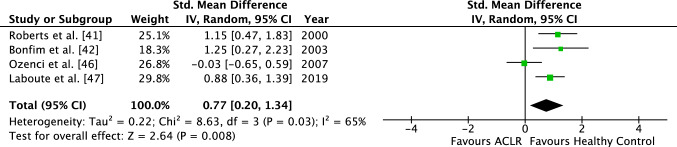
Forest plot of the ACLR group demonstrating the TTDPM between the operated group and the healthy control group. *ACLR* anterior cruciate ligament reconstructed, *TTDPM* threshold to detection of passive motion, *Std.* standardized, *IV* inverse variance, *CI* confidence interval, *df* degrees of freedom

For the four studies analyzing the TTDPM, an SMD of 0.77 (95% CI 0.20–1.34), also favoring the HC group, was identified (Fig. 3). The TTDPM was also significantly higher in the ACLR group compared with the control group (*Z* = 2.64, *p* < 0.01). *I-*squared demonstrated a substantial study heterogeneity with a value of 65% (*p* < 0.05).

### Anterior Cruciate Ligament Deficient (ACLD) versus HCs

**Fig. 4:**
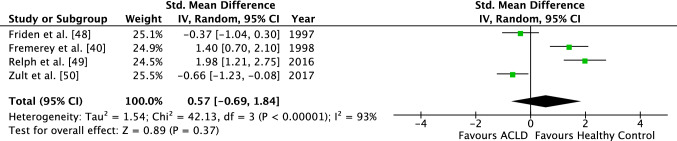
Forest plot of the ACLD group demonstrating the JPS between the conservatively treated group and the healthy control group. *ACLD* anterior cruciate ligament deficient, *JPS* joint position sense, *Std.* standardized, *IV* inverse variance, *CI* confidence interval, *df* degrees of freedom

Across four studies that assessed differences in JPS between ACLD and HC, an SMD of 0.57 (95% CI − 0.69 to 1.84) was identified, which was not statistically significant (*Z* = 0.89, *p* = 0.37). In this comparison, considerable study heterogeneity was demonstrated (*I*^2^ = 93%, *p* < 0.01) (Fig. 4).

**Fig. 5:**
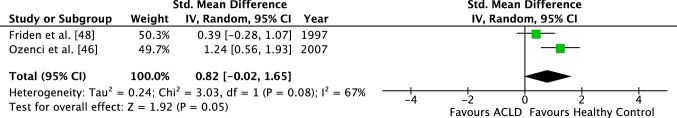
Forest plot of the ACLD group demonstrating the TTDPM between the conservatively treated group and the healthy control group. *ACLD* anterior cruciate ligament deficient, *TTDPM* threshold to detection of passive motion, *Std.* standardized, *IV* inverse variance, *CI* confidence interval, *df* degrees of freedom

An effect size of 0.82 (95% CI − 0.02 to 1.65) was calculated for studies investigating TTDPM. Similarly, no significant difference between the two groups (ACLD vs. HCs) could be found (*Z* = 1.92; *p* = 0.05). The study heterogeneity with an *I-*square of 67% was substantial (*p* = 0.08) (Fig. 5).

### Quality Assessment

Table 5 shows the final quality scores of all included studies, and the mean score. The included studies had a mean quality score of 19 on the Downs and Black Checklist. According to Silverman et al. [34], a score of 19 indicates a sufficient and fair study quality [34].

**Table 5:** Quality score of the included studies assessed using the Downs and Black Checklist

Study	Downs and Black Checklist score
Bonfim et al. [42]	18
Fremerey et al. [40]	20
Fridén et al. [48]	18
Laboute et al. [47]	18
Mir et al. [43]	17
Ozenci et al. [46]	16
Relph et al. [49]	22
Roberts et al. [41]	16
San Martín-Mohr et al. [45]	26
Zhou et al. [44]	17
Zult et al. [50]	17
Mean	19

### Risk of Bias

The assessment of the risk of bias revealed an overall low risk of bias across all studies, with a JBI score rating of between 87.5 and 100% (Electronic Supplementary Material 1). A total of eight studies had a low risk of bias, with all JBI items being met. However, two studies [45, 46] had a high risk of bias regarding strategies to deal with confounding factors. Generally, a high risk of bias might also result from the difficulty of randomization and blinding in such clinical trials including patients.

## Discussion

This systematic review and meta-analysis examined the effects of ACL injury on knee proprioception in patients with surgical arthroscopic ACL reconstruction and conservatively treated patients without surgery compared with HCs. The main findings revealed that proprioception after surgical restoration by anatomic ACL reconstruction was significantly lower compared with the control group, while no significant differences were observed when comparing conservative treatment with HCs. For ACLD patients, outcomes for JPS were more clear compared with the TTDPM (with only two available studies and a *p*-value of 0.05). These results highlight the substantial proprioceptive consequences of surgical ACL repair that need to be taken into account when discussing individuals’ treatment procedures and rehabilitative approaches.

### Proprioceptive and Functional Relevance of an ACL Reconstruction

From a functional perspective, the proprioception of the ACL comprises three major components: (1) a static awareness of the knee joint position; (2) a dynamic detection of knee movement and acceleration; and (3) a closed loop reflex arc, which elicits hamstring reflex responses and regulates synergistic and antagonistic muscle contractions to prevent articular injuries [19, 51, 52]. Adequate sensation of joint movement and joint position in the almost terminal knee position (as observed in this review) is of clinical relevance in conditions when synergistically passive (ligamentous) and active (contractile) restraints to anterior tibial translation or rotational stress are required [24, 53, 54]. The aforementioned conditions entail jumping, pivoting, and landing maneuvers for which scientific evidence suggests that poor proprioception is highly related to motor dysfunction [55, 56] and changes arthrokinematics at articular peak loading in ACL-risky situations [57]. Compromised landing [58] and jumping mechanics [59, 60] arise in unilaterally ACL reconstructed patients compared with HCs and are accompanied by neuromuscular deficits in the musculature encompassing the knee joint [61–63], with the result of an elevated risk of ACL recurrences, and meniscal or cartilage injuries [64, 65]. The delayed detection of disadvantageous knee kinematics when the ACL experiences exceeding strain or strain rates (TTDPM) coupled with inaccuracy in sensory joint positioning are key deficits [referring to (1) and (2) above] that expose ACLR patients to re-injuries [9]. Therefore, adequate assessment of knee proprioception in these vulnerable joint angle positions is of exceptional importance. An additional deficit refers to the delayed and diminished contractile responses [referring to (3) above]: knee extensor and flexor forces account, on average, for up to 4500 N [54] when maximally contracted and therefore serve as important shelters for articular knee structures. In comparison, the ACL only sustains approximately 400 N [66], which is equivalent to a proportion of 1/10 of muscle force. The commonly known quadriceps and hamstring weaknesses after ACL tears [67] are mediated by reduced muscle activations [67] and may arise partly from non-existent stimulation of group II or III fibers due to the surgical removal of mechanoreceptors (synchronously with the ACL) after torn or arthroscopically reconstructed ACL with auto- and allografts [67]. Analyzing the results of this study with reference to the aforementioned literature, it becomes apparent that proprioceptive deficits account for multiple aspects with clinical consequences.

### Proprioceptive and Functional Relevance of an ACL Deficiency

Despite the evidence-based chain of reasoning and functional consequences of an ACL reconstruction, it is under debate why the conservative treatment of an ACL tear is superior compared with ACLR, with regard to proprioception assessed using JPS. The interpretation requires more precautions for TTDPM before an affirmative clinical conclusion can be made as the entire sample size comprises 36 patients only and the level of significance is equal to 0.05. As a further important aspect, the test sensitivity and reliability (for TTDPM > JPS [68]), as well as the required time to recover full proprioceptive capacity after the ACL tear, needs to be considered.

It is generally acknowledged that native and intact ACLs are equipped with mechanoreceptors that are responsible for proper proprioceptive functioning [69]. These receptor types are sensitive to mechanical deformation and modify neuromuscular function by afferent feedback [69]. Indeed, recent studies have demonstrated that the number of mechanoreceptors in the ACL is positively associated with the accuracy of gold-standard measurements (JPS and TTDPM) of proprioception [51]. This indicates that proprioceptive function and functional stability are closely related to the number of mechanoreceptors in ACLD patients with remnants [70]. Remnants are ACL stumps that remain after ligament rupture and have been shown to possess operating mechanoreceptors that seem to be dependent on injury duration [71] and reliant on continued loading of the remnant [72]. The fact that remnants are still equipped with functioning sensory elements led researchers to speculate about their role in proprioception and knee stability in ACLD populations or patients who underwent a recent fusion surgery with the new tendinous graft attached to the remnant [73]. Although the remnant situation was not reported in the majority of included studies in this systematic review, it might be speculated that patients treated with a conservative approach to ACL rehabilitation profit from remaining ACL remnants that again help to preserve knee proprioception, as indicated for the JPS. However, this needs to be further investigated.

### Rehabilitative Aspects and Considerations for Return to Sports

Various models and algorithms have been defined that can guide the rehabilitation and decision for return to sport [74, 75]. The requirements for high-level sports activity after ACL tears are defined as strength, power, balance, proprioception, speed, and agility [76, 77]. Despite proprioception being key among the primary criteria for functional performance and postural stability, there is no consensus on a method to objectively determine the level of proprioception at the time of return to sport after injury. Although previous research demonstrated remarkable correlations between knee proprioception and dynamic balance [78, 79], there is also no consensus about the relative importance of proprioception in the process of decision making for return to sport, especially when considering a net proprioception error of ≤ 2°, as illustrated by the outcomes of the current meta-analysis. Despite the medium to large effect sizes (SMD 0.57–0.82), clinical evidence about the effects of such proprioception deficits on movement kinematics and kinetics is still lacking.

Although not assessed in the current meta-analysis, impaired proprioception in patients with ACLR might facilitate the occurrence of unfavorable knee positions during jumping or landing tasks, which are frequently incorporated in many return to sport protocols [80, 81]. This hypothesis is supported by recent evidence demonstrating clear relationships between knee proprioception and landing kinematics by showing that individuals with higher levels of proprioception were able to better control knee flexion angles at initial contact during dynamic tasks [56].

Since proprioception itself is greatly mediated by neural pathways, holistic strategies of motor control (being greatly reliant on feedback about body and limb position [10]) are moving increasingly into the focus of the multicomponent assessment for ensuring proper recovery from ACLR [82]. As a potential measure, myoelectrical latency and amplitude assessments are often incorporated in return to sport tasks (i.e. frontal, rotational or lateral jumps, running, landing or cutting) and contain important information about the integration of proprioception in sports and the individual protective strategies for injury avoidance [83, 84]. These have to be completed under great time pressure and physical exertion.

### Methodological Considerations

For an adequate interpretation of the results, potential limitations need to be considered. The current systematic review included only studies that assessed proprioception within a time period of 24 months post injury. Although this might impact the generalizability of our findings to longer-lasting proprioceptive deficits, this approach was intended to ensure homogeneity of included studies. Despite the strict inclusion criteria of the present work, there was still heterogeneity between studies, which might impact the interpretation of the results (especially in the comparison between ACL deficient patients and HCs). One potential reason for the observed heterogeneity might be found in the non-standardized protocols for the evaluation of proprioception, including patients’ position (lying, sitting, standing), target angles (10–100°), direction of movements (flexion, extension) and techniques (dynamometer, goniometer, analog scale). To address this heterogeneity, we a priori defined strict eligibility and prioritization criteria. Finally, further research needs to evaluate to what extent these differences lead to functional differences between patients with and without surgical treatment of ACL and HCs. Although the present study focused on studies within a range of 24 months post treatment, it might be speculated that time-dependent changes in proprioception deficits occur for both ACLD and ACLR knees caused by histological healing processes. Since this analysis was not possible within the current design due to limited study availability, future studies are warranted that further investigate this research question.

## Conclusion and Perspectives

The findings from the present systematic review and meta-analysis indicate that proprioceptive deficits are present after operative treatment of ACL. No significant deficits were detected following conservative treatment for JPS; however, greater caution is required in relation to interpretation of TTDPM and drawing an affirmative clinical conclusion. These results are of major importance for clinicians and surgeons when planning an individualized ACL rehabilitation approach. In this context, further research is needed in order to examine the mechanisms underlying the differences for treatment approaches as well as functional relevance and consequences of impaired proprioception in ACL patients. Recent technical progress in ACL surgeries suggests that ACL augmentation using remnant-preserving ACL reconstruction may produce satisfactory clinical outcomes because preservation of the ACL remnant can be beneficial in terms of proprioception, biomechanical functions, and vascularization of the graft. With an emphasis on novel surgical approaches and the duration of healing and rehabilitation periods [85], the interrelationship between surgical reconstruction and the preservation of proprioception needs further elaboration.

## Supplementary Information

Below is the link to the Electronic Supplementary Material.

Supplementary file1 (DOCX 17 KB)

## Declarations

### Availability of data and material

The datasets generated and analyzed during the current systematic review and meta-analysis are available from the corresponding author upon reasonable request.
