# Supplementary material for: Effect of an Anterior Cruciate Ligament Rupture on Knee Proprioception Within 2 Years After Conservative and Operative Treatment: A Systematic Review with Meta-Analysis
Source: Sports Med. 2021 Dec 2;52(5):1091–102. doi: 10.1007/s40279-021-01600-z (PMC9023382; doi:10.1007/s40279-021-01600-z)
Supplement: Supplementary file 1 — Supplementary file1 (DOCX 17 KB) [file 40279_2021_1600_MOESM1_ESM.docx]

**Effect of an anterior cruciate ligament rupture on knee proprioception within two years after conservative and operative treatment – A systematic review with meta-analysis**

**Journal:** Sports Med

# John Dick Fleming^1^, Ramona Ritzmann^2^, Christoph Centner ^1,2^

*^1^ Department of Sport and Sport Science, University of Freiburg, Freiburg, Germany*

*^2^ Praxisklinik Rennbahn, Muttenz, Switzerland*

**Corresponding author:**

Christoph Centner, PhD

University of Freiburg

Department of Sport and Sport Science

Schwarzwaldstraße 175,

79117 Freiburg, Germany

Email: Christoph.centner@sport.uni-freiburg.de

| **Item/Study** | **Fridén et al. 1997** | **Fremerey et al. 1998** | **Roberts et al. 2000** | **Bonfim et al. 2003** | **Ozenci et al. 2007** | **Zhou et al. 2008** | **Mir et al. 2008** | **Relph et al. 2016** | **Zult et al. 2017** | **San Martín-Mohr et al. 2018** | **Laboute et al. 2019** |
| --- | --- | --- | --- | --- | --- | --- | --- | --- | --- | --- | --- |
| **Item 1** |  |  |  |  |  |  |  |  |  |  |  |
| **Item 2** |  |  |  |  |  |  |  |  |  |  |  |
| **Item 3** |  |  |  |  |  |  |  |  |  |  |  |
| **Item 4** |  |  |  |  |  |  |  |  |  |  |  |
| **Item 5** |  |  |  |  |  |  |  |  |  |  |  |
| **Item 6** |  |  |  |  |  |  |  |  |  |  |  |
| **Item 7** |  |  |  |  |  |  |  |  |  |  |  |
| **Item 8** |  |  |  |  |  |  |  |  |  |  |  |

| **Yes (low risk)** |  |
| --- | --- |
| **No (high risk)** |  |
| **Unclear** |  |
| **Not applicable** |  |

**Supplementary Material 1:** Risk of bias for the included studies assessed using the JBI Critical Appraisal Checklist for analytical cross-sectional trials
